# Early sepsis identification following cytoreductive surgery for peritoneal malignancy

**DOI:** 10.1186/s13054-020-2831-9

**Published:** 2020-03-23

**Authors:** Darius Cameron Wilson, Danylo Yershov, Chandrakumaran Kandiah, Nicholas Cortes, Kirsty Gordon, Kordo Saeed

**Affiliations:** 1 Shock, Organ Dysfunction and Resuscitation Research Group, Vall d’Hebron Institut of Research, Barcelona, Spain; 2 South Warwickshire NHS Foundation Trust, Warwick, UK; 3 Peritoneal Malignancy Unit, Basingstoke Hospital, Basingstoke, UK; 4 Gibraltar Health Authority, St Bernard’s Hospital, Gibraltar, UK; 5 Department of Biochemistry, Hampshire Hospitals NHS Foundation Trust, Basingstoke, UK; 6 University Hospital Southampton NHS Foundation Trust, Southampton, UK

Cytoreductive surgery for peritoneal malignancy often involves extensive tissue resection, a prolonged operating time, and a significant post-operative systemic inflammatory response (SIRS) [1]. The development of a subsequent post-surgical infection constitutes a common cause of morbidity and mortality, with intra-abdominal infections, in particular, resulting in high mortality rates due to the progression towards tertiary peritonitis and multiple organ dysfunction [2]. The relatively high incidence of infection development, coupled with the immense systemic inflammatory response generated following surgery, makes an early identification of infection problematic and may result in the suboptimal administration of antibiotics.

Current laboratory tools to aid infection diagnosis following surgery include the use of white cell counts (WCC), C-reactive protein (CRP), and procalcitonin (PCT). However, both WCC and CRP levels can be significantly increased in the absence of infection, whilst post-operative PCT concentrations may be heavily dependent on the type and complexity of the surgery performed [3]. Thus, complementary tools to aid diagnosis are required. Previous studies using mid-regional proadrenomedullin (MR-proADM) have highlighted its potential use as an early marker of sepsis development following severe burn injury [4] due to its involvement in the early stages of capillary leakage, endothelial dysfunction, and multiple organ failure [5, 6]. Accordingly, this biomarker may also be of interest following major surgery.

To test this hypothesis, all patients due to undergo cytoreductive surgery for peritoneal malignancy at the Basingstoke and North Hampshire Hospital were consecutively enrolled between January and December 2017. Kinetic profiles of MR-proADM and CT-proET-1 were compared with those of PCT, WCC, and CRP pre-, intra-, and post-operatively, and for 7 days following surgery.

A total of 50 patients were enrolled with an average operation duration of 7.1 (1.6) h (Table 1). All patients were treated with a combination of Metronidazole and Gentamicin during surgery, whilst 39 (78.0%) were administered vasopressors, 46 (92.0%) treated with intraperitoneal hyperthermic chemoperfusion (HIPEC), and 15 (30.0%) underwent a splenectomy. Furthermore, 15 (30.0%) patients required a blood transfusion during surgery, of which 10 (66.7%) required a follow-up transfusion 3.1 (2.3) days later. A clinical diagnosis of sepsis, defined by the presence of a clinical or radiological infectious focus, a positive pathogen identification and a SOFA score increase of ≥ 2 points, could be confirmed in 4 (8.0%) patients, with 6 (12.0%) additional patients satisfying the same criteria, albeit without a positive pathogen identification. The average time to infection diagnosis was 4.0 (2.1) days following surgery, with additional antibiotics initiated immediately upon diagnosis.

**Table 1:** Patient characteristics and subsequent operative requirements for patients prior to cytoreductive surgery within the total population and infected/non-infected subgroups

Patient characteristics	Total patient cohort (*N* = 50)	Non-infected patients (*N* = 40)	Infected patients (*N* = 10)	*p* value
Demographics				
Age (years) (mean, SD)	60.4 (13.1)	60.8 (12.7)	59.5 (15.9)	0.802
Male gender (*N*, %)	24 (48.0%)	20 (50.0%)	4 (40.0%)	0.127
BMI (kg/m^2^) (mean, SD)	28.5 (5.5)	28.4 (5.6)	29.0 (5.0)	0.763
Disposition				
Hospital duration (days) (median, Q1–Q3)	16.5 [13 – 20]	15.5 [12 – 19]	21 [17.25 – 22]	0.112
ITU duration (days) (median, Q1–Q3)	2 [1.25 – 3]	2 [1 – 3]	2.5 [2 – 6]	0.288
Comorbidities				
Cardiovascular (*N*, %)	13 (26.0%)	11 (27.5%)	2 (20.0%)	0.873
Respiratory (*N*, %)	3 (6.0%)	3 (7.5%)	0 (0.0%)	0.119
Immunodeficiency (*N*, %)	0 (0.0%)	0 (0.0%)	0 (0.0%)	1.00
Diabetes (*N*, %)	3 (6.0%)	3 (7.5%)	0 (0.0%)	0.117
Renal (*N*, %)	0 (0.0%)	0 (0.0%)	0 (0.0%)	1.00
Liver (*N*, %)	2 (8.0%)	2 (5.0%)	0 (0.0%)	0.274
Central nervous system (*N*, %)	2 (8.0%)	2 (5.0%)	0 (0.0%)	0.288
Operative requirements				
Operation duration (h) (median, Q1–Q3)	7 [5.7 – 8.1]	6.7 [5.7 – 7.6]	8.7 [7.3 – 9.4]	0.035
HIPEC (*N*, %)	46 (92.0%)	36 (90.0%)	10 (100.0%)	0.782
Splenectomy (*N*, %)	15 (37.5%)	8 (20.0%)	7 (70.0%)	0.001
Vasopressors (*N*, %)	39 (78.0%)	33 (82.5%)	6 (60.0%)	0.571
Biomarkers				
MR-proADM (nmol/L) (median, Q1–Q3)	0.59 [0.47 – 0.73]	0.61 [0.48 – 0.75]	0.56 [0.42 – 0.64]	0.983
CT-proET-1 (pmol/L) (median, Q1–Q3)	63.1 [49.4 – 70.8]	63.9 [49.4 – 67.8]	62.2 [50.3 – 71.8]	0.984
PCT (ng/mL) (median, Q1–Q3)	0.05 [0.03 – 0.07]	0.05 [0.03 – 0.07]	0.05 [0.03 – 0.09]	0.179
CRP (mg/L) (median, Q1–Q3)	12 [7 – 18]	12 [7 – 22]	11 [6 – 18]	0.689
WCC (10^9^/L) (median, Q1–Q3)	8.0 [6.3 – 11.1]	7.9 [6.0 – 11.6]	8.0 [6.2 – 12.3]	0.816

Patients were classified into subgroups based on post-surgical infection development (infected vs. non-infected: *N* = 10 vs. *N* = 40; Table 1), with no differences in any biomarker concentration at pre-surgical or intra-surgical time points, or for CT-proET-1 in the subsequent days following surgery (Fig. 1). Significant differences, however, were found between both groups for MR-proADM, PCT, CRP, and WCC at varying time points. MR-proADM concentrations were significantly elevated 1 day (T + 1) after surgery and at all subsequent time points thereafter (infected vs. non-infected: 2.2 [1.5 – 2.5] vs. 1.2 [1.0 – 1.4] nmol/L; *p* < 0.001), whereas PCT concentrations were significantly elevated 2 days (T + 2) after surgery (infected vs. non-infected: 3.1 [1.4 – 4.5] vs. 0.7 [0.3 – 1.8] ng/mL; *p* < 0.01). Both CRP and WCC were only significantly elevated at time point T + 3 (*p* < 0.01). Corresponding AUROC analysis for MR-proADM at T + 1 was 0.90 [0.81 – 1.0] (cut-off: 1.96 nmol/L; sensitivity: 0.90 [0.60 – 0.98]; specificity: 0.85 [0.71 – 0.93]), whereas PCT at T + 2 was 0.77 [0.63 – 0.92] (cut-off: 0.68 ng/mL; sensitivity: 1.0 [0.72 – 1.0]; specificity: 0.48 [0.33 – 0.63]).

**Fig. 1:**
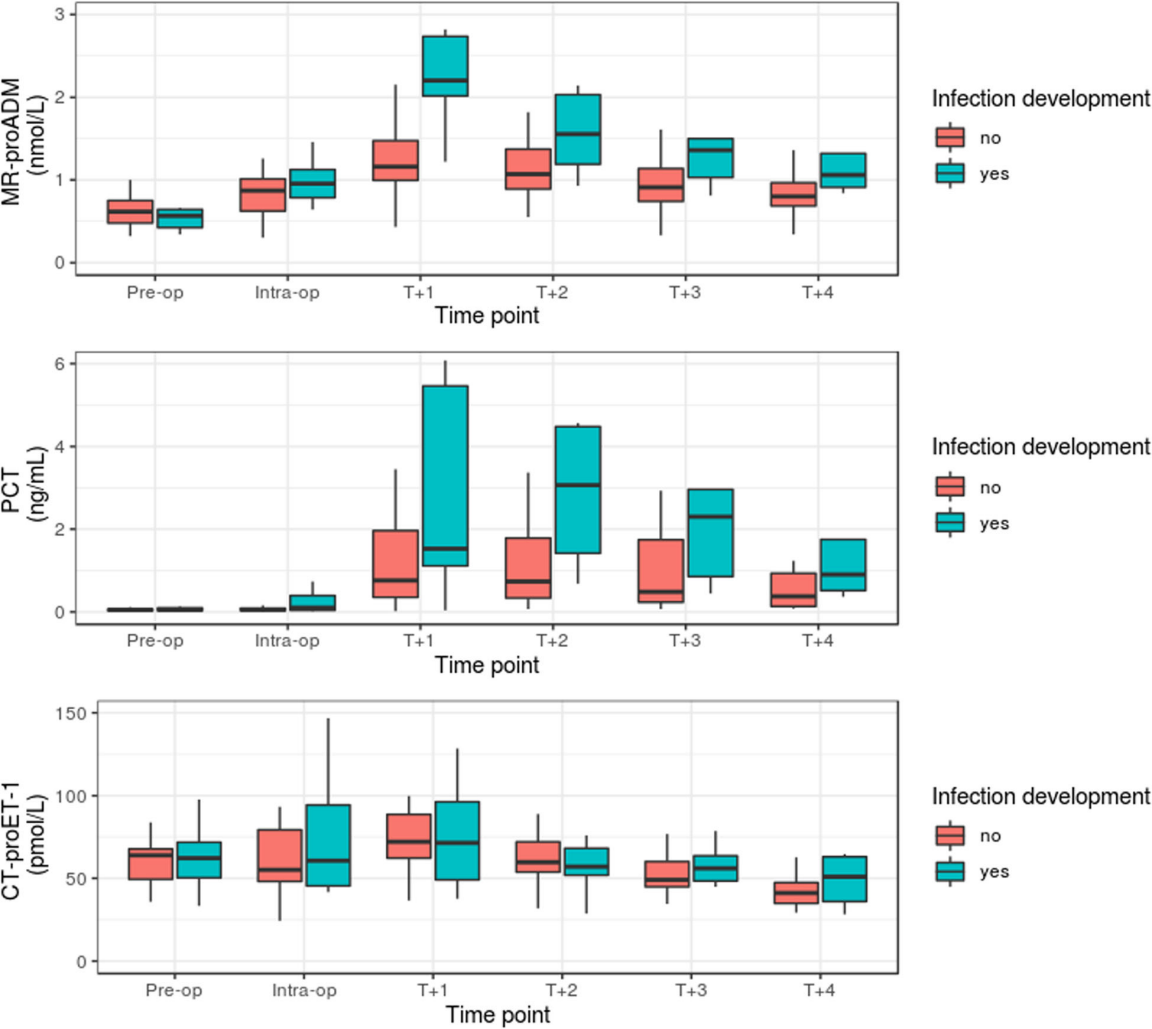
Kinetic profiles of MR-proADM, PCT, and CT-proET-1 biomarkers before, during, and after cytoreductive surgery for peritoneal malignancy. Analysis of the effect of time and infection development on PCT and MR-proADM concentrations in a 2-way ANOVA found significant effects for both factors as well as for the interaction for MR-proADM (*p* < 0.001), indicating that MR-proADM values are not only modulated over time and show an offset between infected and uninfected patients, but that the time course of MR-proADM has a different trajectory for both patient groups. PCT, on the other hand, also showed significant effects for both factors (*p* < 0.01), although no significant interaction could be found (*p* = 0.77), indicating no trajectory differences over time between both patient groups

Results indicate that MR-proADM kinetics are increased earlier and are more accurate than PCT in identifying patients at risk of developing an infection after cytoreductive surgery for peritoneal malignancy. Additional studies with a larger sample size are required to confirm these hypothesis-generating findings.

## Data Availability

All relevant datasets are available from the corresponding author upon reasonable request.

## Abbreviations

ANOVA: Analysis of variance
AUROC: Area under the receiver operating characteristic curve
CRP: C-reactive protein
CT-proET-1: C-terminal proendothelin-1
HIPEC: Hyperthermic chemoperfusion
MR-proADM: Mid-regional proadrenomedullin
PCT: Procalcitonin
SIRS: Systemic Inflammatory Response Syndrome
WCC: White cell count
